# A first-in-class inhibitor of HSP110 to potentiate XPO1-targeted therapy in primary mediastinal B-cell lymphoma and classical Hodgkin lymphoma

**DOI:** 10.1186/s13046-024-03068-x

**Published:** 2024-05-22

**Authors:** Manon Durand, Vincent Cabaud Gibouin, Laurence Duplomb, Leila Salmi, Mélody Caillot, Brigitte Sola, Vincent Camus, Fabrice Jardin, Carmen Garrido, Gaëtan Jego

**Affiliations:** 1 INSERM, UMR1231, Team HSP-Pathies Labellisée « Ligue Nationale Contre Le Cancer » and Labex LipSTIC, Dijon, 21000 France; 2 University of Burgundy, Medical Sciences Faculty, Dijon, 21078 France; 3 INSERM, UMR1231, Equipe GAD, University of Burgundy, Dijon, 21078 France; 4 INSERM, U1245, Normandy University, Caen, 14000 France; 5 Department of Hematology, Centre Henri Becquerel, Rouen, 76000 France; 6 Georges François Leclerc Cancer Centre, CGFL, Dijon, France; 7 INSERM, UMR1231, Université Bourgogne, 7 Boulevard Jeanne d’Arc, Dijon, 21078 France

**Keywords:** Heat-shock protein, XPO1, STAT6, Lymphoma

## Abstract

**Background:**

Primary mediastinal B-cell lymphoma (PMBL) and classical Hodgkin lymphoma (cHL) are distinct hematological malignancies of B-cell origin that share many biological, molecular, and clinical characteristics. In particular, the JAK/STAT signaling pathway is a driver of tumor development due to multiple recurrent mutations, particularly in STAT6. Furthermore, the *XPO1* gene that encodes exportin 1 (XPO1) shows a frequent point mutation (E571K) resulting in an altered export of hundreds of cargo proteins, which may impact the success of future therapies in PMBL and cHL. Therefore, targeted therapies have been envisioned for these signaling pathways and mutations.

**Methods:**

To identify novel molecular targets that could overcome the treatment resistance that occurs in PMBL and cHL patients, we have explored the efficacy of a first-in-class HSP110 inhibitor (iHSP110-33) alone and in combination with selinexor, a XPO1 specific inhibitor, both in vitro and in vivo.

**Results:**

We show that iHSP110-33 decreased the survival of several PMBL and cHL cell lines and the size of tumor xenografts. We demonstrate that HSP110 is a cargo of XPO1^wt^ as well as of XPO1^E571K^. Using immunoprecipitation, proximity ligation, thermophoresis and kinase assays, we showed that HSP110 directly interacts with STAT6 and favors its phosphorylation. The combination of iHSP110-33 and selinexor induces a synergistic reduction of STAT6 phosphorylation and of lymphoma cell growth in vitro and in vivo. In biopsies from PMBL patients, we show a correlation between HSP110 and STAT6 phosphorylation levels.

**Conclusions:**

These findings suggest that HSP110 could be proposed as a novel target in PMBL and cHL therapy.

**Supplementary Information:**

The online version contains supplementary material available at 10.1186/s13046-024-03068-x.

## Background

Primary mediastinal B-cell lymphoma (PMBL) is a rare and distinct entity of aggressive large B-cell lymphoma (LBCL) of thymic B-cell origin that is typically present as a large anterior mediastinal mass and predominantly affects women in their thirties [1]. Classical Hodgkin lymphoma (cHL) is a rare but highly curable malignancy preferentially affecting adolescents or young adults and those over 60 [2, 3]. cHL cells originate from germinal center-derived B cells (Hodgkin and Reed–Sternberg (HRS) cells) surrounded by an infiltrate with abundant immune cells [4]. Mediastinal involvement is also a typical presentation of nodular sclerosis classical Hodgkin lymphoma (NSCHL), which is the most common type of cHL. PMBL shares strong biological, molecular and clinical characteristics with cHL, particularly NSCHL [5, 6]. Indeed, the central role played by the JAK/STAT signaling pathway in tumor cell viability is a common feature. In both diseases, amplification of the JAK/STAT signaling pathway is due to mutations in STAT6 genes [6, 7], and multiple recurrent mutations of genes encoding regulatory molecules like *SOCS1* (suppressor of cytokine signaling 1) and *PTPN1* [8, 9]. JAK2 expression and activity is also increased in PMBL and cHL because of chromosome 9p24.1/JAK2 amplification [10, 11]. NFkB signaling is also amplified in both lymphomas [12] as the most frequently mutated proteins in this pathway, in PMBL [13] and in cHL [7, 14, 15], are TNFAIP3 (tumor necrosis factor, a-induced protein 3), NFKBIE (NFKB inhibitor ɛ), NFKB2 (NFKB subunit 2) and IKBKB (inhibitor of NFKB kinase subunit β).

Although both lymphomas have good prognosis with a survival rate exceeding 80% at 5 years [4, 16] about 20% of patients are refractory after first-line treatment or relapse early, with consequent suboptimal outcomes. Identification of novel molecular targets would therefore be a way to develop future tailored therapies to overcome these resistances. In 25% of PMBL and cHL, the *XPO1* gene, which encodes exportin 1 (XPO1), which then controls the nuclear export of cargo proteins and RNAs, harbors a recurrent *XPO1* point mutation (NM_003400, chr2:g61718472C > T) resulting in the E571K substitution within the site of cargo binding. The XPO1 exportome is estimated to contain up to 1000 unique cargo molecules, including NFkB and STAT signaling members [17–21]. This exportome is altered by the E571K mutation and could favor disease progression [22–24]. Several exportin inhibitors targeting XPO1 have thus been developed, including selinexor [25]. Because PMBL and cHL cells are sensitive to selinexor, a drug combination including selinexor could potentially be used as a second-line treatment [25, 26].

Heat shock proteins (HSPs), which are molecular chaperones with unique cytoprotective properties, are overexpressed by tumor cells, including in hematological malignancies [27–29]. HSPs have a role in the correct folding, activity, transport, and stability of proteins, thereby assuring cell survival. Tumor cells depend heavily on HSPs because of their capacity to sustain the high rate of protein synthesis, folding and overall metabolism. Among many functions, they are strongly associated with key oncogenes such as BCR-ABL, FLT3-ITD fusion proteins in leukemias or EGFR in breast cancer and promote overactivated signaling pathways [28]. However, very few studies have documented the role of intracellular HSPs in cHL [27], and none have been conducted in PMBL [27]. Among the different HSPs, there has been a renewed interest in the long-forgotten high-molecular-weight HSP110 since the discovery of an inactivating mutation in colorectal cancer associated with an excellent prognosis [30]. But HSP110 also appears to be an important player in diffuse large B-cell lymphoma (DLBCL) survival as it correlates with the aggressiveness and proliferation index in patients [31]. HSP110 expression correlates with c-MYC, BCL6 and MYD88 protein expression in tumor biopsies of DLBCL, and HSP110 siRNA-mediated knockdown leads to decreases in these oncogenes and cells survival [32, 33]. Therefore, HSP110 appears to be a new therapeutic target in DLBCL. In this context, our recent identification of small chemical-specific inhibitors of HSP110 (iHSP110) has opened the way to new combinational drug testing [34]. Here, we explored the expression of HSP110 and sensitivity of PMBL and cHL cells to the HSP110-specific inhibitor iHSP110-33 alone or in combination with selinexor. We show that the treatment of PMBL and cHL cell lines with these compounds alone decreased their survival, and that using the inhibitors in combination leads to a synergistic effect in vitro and in vivo. Our assessment of HSP110-STAT6-XPO1 interactions at the molecular level revealed that HSP110 is a cargo of XPO1 independently of its mutational status. HSP110 directly interacts with STAT6 and facilitates it phosphorylation, and this effect is blocked by iHSP110-33 alone and even more so when combined with selinexor. Furthermore, we show a correlation between HSP110 (both mRNA and protein) and STAT6 phosphorylation in PMBL patients.

## Methods

### Primary tumors and cell lines

The PMBL cell lines K1106P (XPO1^wt duplicated^), MedB1 (XPO1^wt duplicated/E571K^) and U2940 (XPO1^wt^) were obtained from DSMZ. PMBL cell lines KAS, KS and the cHL cell lines L428 (XPO1^wt amplified^), L1236 (XPO1^wt amplified/E571K^), HD-MY-Z (XPO1^wt^) and SUP-HD1 (XPO1^wt/E571K^) were generously provided by Dr. Brigitte Sola (University of Caen, Normandy, France). The KAS (antisense orientation) and KS (sense orientation) clones derived from U2940 express a wild-type allele and a mutated allele C528S/E571K. C528S is a mutation in XPO1 that confers resistance to selinexor, and E571K is a mutation that alters the localization and interactome of XPO1. The genetic modification strategy used is CRISPR-Cas9, as described previously [19]. HEK293 STAT6-mutant cell line [35] was graciously supplied by Pr. Karen Leroy (European Georges-Pompidou Hospital, Paris Descartes University, France) and was stimulated with IL-4 (130–093-919, Miltenyi Biotec, Bergisch Gladbach, Germany). To establish the mutant cell line, HEK293^WT^ cell line was transfected with the expression vector pcDNA3.1 (Invitrogen) containing the coding sequence of STAT6 in the vector's multiple cloning site. The coding sequence of STAT6 was obtained by PCR amplification using cDNA from Ramos and MedB-1 cell lines. Subsequently, cells were selected using 800 µg/mL of G418 (Invitrogen). Transfected cells are maintained in culture with 600 µg/mL of G418. K1106P, and MedB1 were cultured in IMDM (P04-20150, PAN Biotech, Aidenbach, Germany), supplemented with 20% FBS (P04-96650, PAN Biotech). U2940, L428, and L1236 cells were cultured in RPMI 1640 (L0500-500, Dutscher group, Saint-Cyr-L'Ecole, France), supplemented with 10% FBS. U2940 and L1236 culture media were supplemented with 1% non-essential amino acid solution (100x) (Fisher, Hampton, USA). HEK-293 cells were cultured in DMEM (L0060-500, Dutscher group) with 10% FBS. Tumor samples for immunohistochemistry (IHC) and Proximity Ligation Assay (PLA) came from the PMBL LYSA cohort, a multicenter retrospective study that assessed the clinical outcomes of previously untreated PMBL patients who received first-line immunochemotherapeutic treatment [36]. Available formalin-fixed, paraffin-embedded (FFPE) tissue blocks obtained at the time of initial diagnosis were collected and centrally reviewed for PMBL diagnosis confirmation by expert hematopathologists following the diagnostic criteria established by previous pathologic descriptions of PMBL from the literature and international classifications [37–39]. After molecular characterization of these cases, as recently reported by Camus et al. [40], the remaining available samples from confirmed PMBL cases were provided by the coordinating investigator and used for the present work. The utilization of human biopsies in our research received approval from the relevant institutional review boards or ethics committees, and all human participants provided informed consent.

#### Reagents and inhibitors

iHSP110-33 (SYNTHENOVA SAS, Hérouville-Saint-Clair, France) is a functional inhibitor of HSP110, that binds to the nucleotide-binding domain of HSP110 [34]. iHSP110-33 was solubilized in DMSO at 30 mM, aliquoted for single use, and stored at -80 °C. Selinexor (KPT-330) (SelleckChem, Houston, TX, USA) was solubilized in DMSO at a concentration of 100 mM, aliquoted for single use, and stored at -80 °C. Selinexor specifically binds to XPO1 leading to the inhibition of nuclear export. MG-132 (Sigma Aldrich, M7449, Merck, Rahway, NJ, USA) is a ready-to-use solution solubilized in 200 µL of absolute ethanol, aliquoted for single use, and stored at -20 °C. MG132 is a strong inhibitor of proteins degradation through the proteasome. Recombinant human interleukin-4 (Miltenyi Biotec, 130–093-919), is a ready-to-use solution at 50 µg/mL, aliquoted for single use, and stored at -20 °C.

### Transfection

PMBL and cHL cell lines were transfected using the AMAXA Nucleofector 2b device (Lonza, Basel, Switzerland) and the corresponding Nucleofector kit: T for K1106P, V for MedB1, U2940 and L1236, L for L428. Transfections were carried out with either 1 nmol of siRNA control (10025994, Fisher Scientific) or 1 nmol of siRNA targeting HSPH1 (10584615, Thermo-Fisher Scientific, Waltham, MA, USA). Specific AMAXA programs were applied: 0–020 for K1106P, X-005 for MedB1, and U2940, X-001 for L428, and T-001 for L1236. For HEK293 WT and HEK293 STAT6 cells, transfection was performed using the FuGENE HD Transfection Reagent (E2311, Promega, Madison, WI, USA). The HSP110-GFP plasmid used was homemade**.** XPO1wt-mCherry plasmid and XPO1 E571K-mCherry plasmid were previously described [26].

### Western blots and co-immunoprecipitation

Cells were washed in PBS and lysed on ice a in lysis buffer (9803S, Cell Lysis Buffer, Sigma-Aldrich, Lyon, France) in the presence of protease inhibitors (11836145001, MERCK) and phosphatase inhibitors (P5726, P0044, Sigma-Aldrich, Saint-Louis, MI, USA). Proteins were separated by SDS-PAGE following standard protocols with precast gels (Biorad, Hercules, CA, USA) and transferred with Trans-Blot® Turbo™ Transfer System (Biorad) before analysis with a chemiluminescence detection kit (1705062, 1705061, Biorad). The primary antibodies used were: anti-STAT6 (5397S), -pSTAT6 (9361S), -XPO1 (46249S), -HSP60 (12165S), -RelA (8242S), -TBP (D5C9H), -HSP110 (sc74550) and -GAPDH (sc-47724) from Santa Cruz Biotechnology (Dallas, TX, USA), -Vinculin (V9131, Sigma-Aldrich), -GFP (ab290, Abcam, Cambridge, UK), mCherry (ab213511, Abcam), -Lamin A (MA3-1000, Thermo-Fisher Scientific). The anti-rabbit IgG HRP-linked antibody (7074S) and anti-mouse IgG HRP-linked antibody (7076S) were purchased from Cell signaling Technology (Danvers, MA, USA). Dilutions of the antibodies used for western blot are provided in supplementary Table 1. Cytoplasmic and nuclear extracts were obtained using the NE-PER™ Nuclear and Cytoplasmic Extraction Reagent Kit (78833, Thermo-Fisher Scientific).

Immunoprecipitation was performed using MACS® Technology (µ Columns 130–042-701, µMACS™ Protein A/G MicroBeads 130–071-001, Milteniy) with anti-STAT6 (5397S) and -XPO1 (46249S) antibodies from Cell signaling Technology, -HSP110 (ab108625), -GFP (ab1218), and -mCherry (ab213511) from Abcam.

### Immunohistochemistry and Duolink® proximity ligation assay (PLA)

Cell lines were fixed using 4% paraformaldehyde in PBS, permeabilized using 100% chilled methanol, and blocked using 0.1% Tween-20 in Tris-buffered saline with 3% bovine serum albumin. For PLA on cell lines, the following primary antibodies were used: anti-HSP110 (sc-74550, Santa Cruz Biotechnology), -HSP70 (ADI-SPA-812, Enzo Life Sciences, Farmingdale, NY, USA), -STAT6 (5397S, Cell Signaling Technology), -XPO1 (46249S, Cell Signaling Technology), -BRCA1 (PLA0185, Sigma-Aldrich)**.** Duolink® experiments were performed following the manufacturer's instructions (Sigma-Aldrich) and mounted with ProLong Gold medium with DAPI (4',6-diamidino-2-phenylindole) (P36935, Thermo-Fisher Scientific). For PLA on formalin-fixed and paraffin-embedded tissues, antigens were unmasked (ab208572, Abcam) and endogenous peroxidase was inhibited by 3% hydrogen peroxide in PBS. The Duolink® PLA brightfield (DUO92012, Sigma-Aldrich) was used. IHC staining was performed with Vector NovaRED® Substrate Kit, peroxidase (HRP, SK-4800, Vector Laboratories, Newark, CA, USA). Tissues were counterstained with Harris hematoxylin (Sigma-Aldrich). For fluorescent Duolink® experiments (DUO9201, Sigma-Aldrich), tissues were incubated with 0.1% Sudan Black B (199664, Sigma-Aldrich) to quench lipofuscin autofluorescence after reaction, and with DAPI for nuclear staining. Finally, the slides were mounted using Fluoromount-G (15586276, Thermo-Fisher Scientific). Images were captured using an Axio Imager 2 (Carl Zeiss Microscopy GmbH) with an AxioCam MRm CCD camera (Carl Zeiss Microscopy GmbH) and analyzed with the spot detector plugin for ICY software.

### Image analysis and spot counting

Microscopy images were acquired using multidimensional acquisition with the Axioscope for IHC and PLA brightfield images and the Axio Imager 2 for fluorescence images (Carl Zeiss Microscopy GmbH, Jena, Germany) with a × 40 objective lens. For each tissue section, 3 images were captured at different locations. In the PLA experiment, images were then stacked (z-stack) in ImageJ software and separated into RGB. The blue and red channels were merged for fluorescent images, while the green channel was retained for brightfield images, and then saved in TIFF format. In the ICY software, the "spot detector" plugin was applied to TIFF images with a sensitivity set at scale 2 (3 pixels). Statistical analysis was conducted using GraphPad Prism V8 (Kruskal–Wallis test, with at least 100 cells counted for each condition in fluorescent analysis). For the quantification of IHC images, ImageJ software was used. The "color deconvolution" plugin was employed to determine the ROIs of the 3 staining colors: Harris hematoxylin violet, Nova Red red, and background gray. Eight pixels were selected for each color. The images were then split based on these 3 ROIs. Thresholding was adjusted using the "Moments" program, and then the percentage of area covered by the 3 colors was automatically measured for each image using a programming macro. The staining was normalized to hematoxylin, and the correlation curve was generated using GraphPad Prism 8 software (Spearman coefficient calculated with a confidence level (Cl) of 0.05, *p*-value < 0.01).

### Cell survival and cell proliferation

CellTiterGlo 2.0 Cell Viability experiments and Cell Trace Violet (C34571) were performed following the manufacturer’s instructions (Thermo-Fisher Scientific). For flow cytometry, cells were washed with cold PBS and incubated at 4 °C for 15 min with Annexin-V-FITC (550475, BD Biosciences, Franklin Lakes, NJ, SA). Finally, 7-aminoactinomycin D (7AAD, 5559925, BD Biosciences) was added a few minutes prior to acquisition using a BD LSR Fortessa flow cytometer and analyzed with FlowJo V10.

### Chorioallantoic membrane (CAM) assay

Fertilized eggs (EARL Les Bruyères, Dangers, France) were incubated at 37.5 °C and 60% humidity for 9 days. Eggs were then sterilized with 70% ethanol solution, opened at the center of the eggshell, and inoculated directly on the CAM with 2 × 10^6^ cells in 50 µL of 50% Cultrex Basement Membrane Extract, Type 3, Pathclear (3632–010-02, Bio-Techne, Minneapolis, MN, USA) and 50% of medium without FBS. The window was sealed with invisible tape and the eggs were incubated at 37 °C. On days 12, 14, and 16, the tumors were treated with HSP110 inhibitor, selinexor (KPT-330) or the control solvent DMSO, which was diluted in culture medium. On day 17, the eggs were removed from the incubator and chilled on ice for 1 h. Tumors were then extracted from the CAM for mass measurement, western blotting, and immunohistochemistry (IHC).

### Microscale thermophoresis

Microscale thermophoresis was conducted using a Monolith NT.115 device (Nanotemper, Munich, Germany) to detect the binding affinity between His-tagged HSP110 (TP307102, OriGene, Rockville, MD, USA) and STAT6 (ab125625, abcam) following the manufacturer's instructions. Recombinant His-Tagged HSP110 was labeled using the His-Tag non-covalent labelling kit RED-Tris-NTA 2nd generation (MO-L018, Nanotemper). The experiment was conducted with a fixed His-tagged HSP110 concentration of 50 nM, and the concentration range of STAT6 starting from 2.39 µM to 0.07 nM. The software used was MO.Control 1.6.1 and the analysis was performed with MO.Affinity Analysis v2.3.

### In vitro phosphorylation assay

Recombinant active JAK2 protein (50 ng, 14–640, MercK) was added to the kinase reaction buffer (9802, Cell Signaling Technology) in the presence or absence of 100 ng of recombinant STAT6 (ab125625, Abcam), 100 ng of recombinant HSP110 (TP307102, OriGene), 300 µM HSP110 inhibitor (Synthenova), and 250 µM ATP (9804, Cell Signaling Technology). After incubation for 15 min at 30 °C in a final reaction volume of 40 µL, Laemmli's buffer was added to stop the reaction. STAT6 phosphorylation was then determined by immunoblotting analysis as described previously [41].

### RNA extraction and real-time RT-qPCR

Following deparaffinization, total RNA samples were extracted from two 20-µm FFPE full sections using the Maxwell 16 system (Promega, Manheim, Germany) or, when available, from frozen tissues using the RNA NOW kit (Biogentex, Seabrook, TX) according to the manufacturer’s instructions, then stored in nuclease-free water at -80 °C. cDNA were generated from 200 ng of RNA using Reliance select cDNA synthesis kit (#12012802)(Bio-Rad). Real-time PCR was performed in triplicate in 384-well plates, using a CFX96™ device (Bio-Rad). Briefly, 4 ng (2 µL) of cDNA were mixed with 0.5µL of the primers/probe mix, 5 µL of iTaq Universal probe mix (#1725132) (Bio-Rad) and 2.5 µL of nuclease-free H_2_O. Thermal cycling protocol was: 30 s at 95 °C, followed by 40 cycles of 10 s at 95 °C, 30 s at 60 °C. Analyses were performed using CFX manager software (Bio-Rad).

### Drug combination analysis

For synergy analysis, relative cell viability measurements were assessed using the CellTiterGlo 2.0 Cell Viability assay and averaged (*n* = 3) for all combinations of iHSP110 and selinexor concentrations. Luminescent intensity readings were normalized to the average of control wells on the same plate to obtain relative cell viability values. Synergy summary scores were calculated by averaging viability scores across the entire dose–response landscape. Drug synergism was assessed using the Bliss Independence Model. Bliss synergy scores were computed using SynergyFinderV2 online web application tool (http://synergyfinder.fimm.fi/) [42, 43] and visualized using GraphPad Prism 8 software. Statistical analysis was conducted using two way ANOVA with CI: 0.05 and *p*-value < 0.0001.

## Results

### HSP110 inhibition reduces PMBL and cHL cells growth in vitro and in vivo

**Fig. 1:**
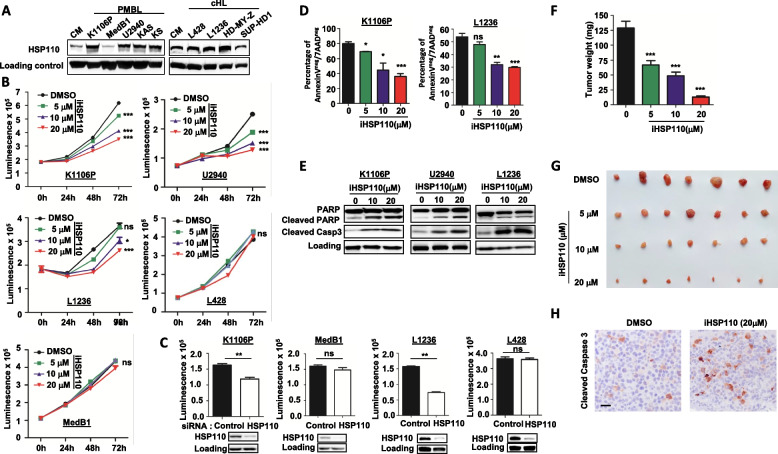
HSP110 specific inhibition, reduces PMBL and cHL growth in vitro and in vivo. **A** Immunoblot analysis of HSP110 in PMBL cell lines (K1106P, MedB1, U2940, U2940-derived KAS, U2940-derived KS), and cHL cell lines (L428, L1236, HD-MY-Z, SUP-HD1). **B** In vitro cell growth of K1106P, U2940, L1236, L428, and MedB1 after 24 h, 48 h, and 72 h of treatment with increasing concentrations the HSP inhibitor (iHSP110-33), measured by CellTiter-Glo. **C** In vitro cell growth of K1106P, MedB1, L1236, and L428 48 h after transfection with an siRNA targeting HSP110 or a control siRNA, measured by CellTiter-Glo. Transfection was validated by immunoblot analysis. B-actin served as a loading control. **D** Survival of K1106P and L1236 measured by Annexin-V/7AAD staining after 72 h of treatment with increasing concentrations of iHSP110. **E** Immunoblot analysis of PARP, cleaved PARP, and cleaved Caspase 3 in K1106P, U2940, and L1236 after 72 h treatment with 10 or 20 µM of iHSP110. B-actin served as a loading control. **F** Treatment with iHSP110-33 induces K1106P tumor mass reduction in the CAM of 17-day-old embryos. Tumor weight of K1106P tumors in CAM treated with iHSP110-33 (5, 10, and 20 µM), or DMSO as a control was measured 7 days after xenograft. Mean mass (± SD) is represented (*n* = 7 per group). **G** Ex vivo images of representative K1106P xenografts as in (**G**). **H** Immunohistochemical images of cleaved Caspase 3 in K1106P tumor xenografts as in Fig. 1F, scale bar 40μm. ns *P* > .05; * *P* < .05; ** *P* < .01; *** *P* < .001; **** *P* < .0001

HSP110 protein expression was first determined in several PMBL and cHL cell lines by western blot. Given the importance of XPO1^E571K^ in the biology of PMBL and cHL, we added two U2940 derived cell lines to our panel, KS and KAS, which were modified to bear the sense and antisense XPO1 double mutants, respectively, as previously published [19]. HSP110 protein expression was heterogeneously expressed by all cell lines irrespectively of XPO1 mutation (Fig. 1A). To determine the impact of HSP110 inhibition on cell growth and survival, we treated several PMBL and cHL cell lines with a HSP110-targeting compound that we screened from a library of foldamers. This compound was selected for its capacity to specifically inhibit HSP110 without altering HSP110 protein levels (supplementary Fig. 1A), and we called it iHSP110-33 [34]. We observed a decrease in cell growth at as early as 48 h in the PMBL cell lines K1106P, U2940, but not in MedB1, and in the cHL cell line L1236 but not in L428, in a dose-dependent manner (Fig. 1B). We confirmed that the effect on the molecule reducing the growth of these cells involved HSP110 by down expressing HSP110 using siRNA (Fig. 1C). The decreased in cell growth upon HSP110 inhibition could be explained by reduced cell survival (Fig. 1D). Apoptosis was confirmed by PARP and caspase-3 cleavage in responsive cell lines (Fig. 1E). A reduced proliferation rate was also observed in K1106P in which HSP110 was depleted by means of a siRNA (supplementary Fig. 1B). To confirm the importance of HSP110 expression in PMBL and the efficacy of the HSP110 inhibitor in vivo, we performed K1106P xenografts on chick embryo chorioallantoic membranes. We observed tumor growth inhibition of 47% at 5 µM, 62% at 10 µM and of 91% at 20 µM in the iHSP110-33-treated embryos compared to the control arm (i.e. DMSO alone. Fig. 1F and G). Increased of cleaved caspase 3 was also observed in isolated tumors treated with iHSP110-33 in vivo (Fig. 1H). Taken together, these data show that HSP110 can be a valuable target for some PMBL and cHL and that our HPS110 inhibitor is effective both in vitro and in vivo.

### HSP110 is a cargo protein of XPO1

**Fig. 2:**
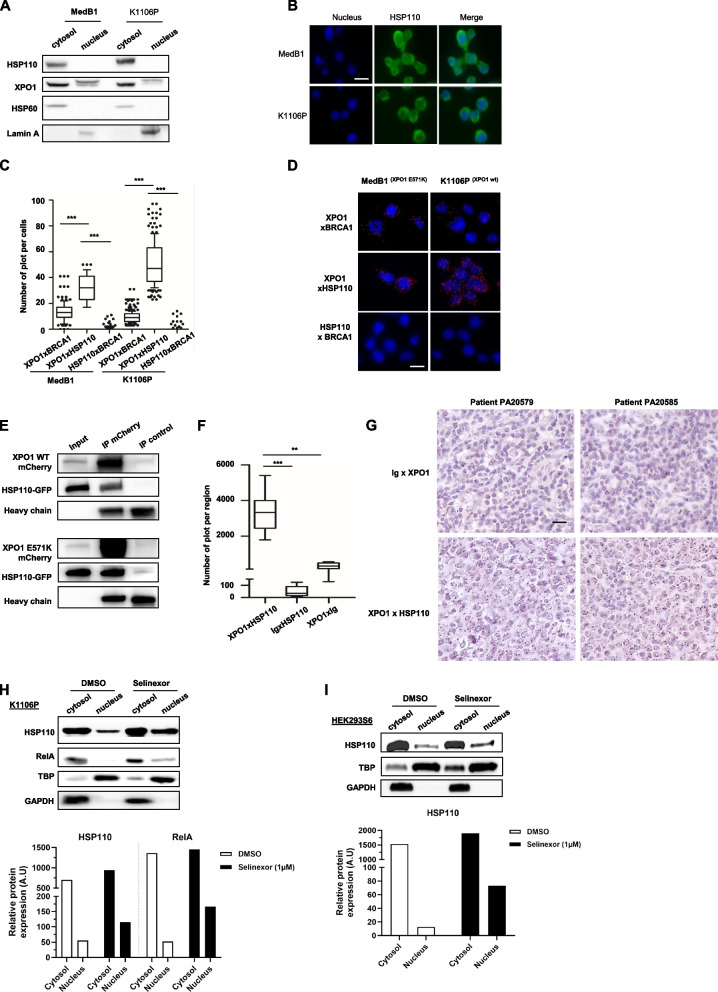
HSP110 is a cargo of XPO1. **A** Immunoblot analysis of HSP110 and XPO1 in the nucleus and in the cytosol of MedB1 and K1106P cells. HSP60 was used as a cytosol loading control, and Lamin A as a nuclear loading control. **B** Immunostaining of HSP110 in K1106P and MedB1 cells. Cell nuclei were stained with DAPI. Scale bar: 10 µm **C** Quantification of the interactions of XPO1-BRCA1, HSP110-BRCA1, and XPO1-HSP110 in MedB1 and K1106P cells using Duolink technology. BRCA1 was used as a negative control for HSP110 and XPO1 interactions. ***P < .001. **D** Representative images of the interactions of XPO1-BRCA1, HSP110-BRCA1, and XPO1-HSP110 in MedB1 and K1106P cells as in (**D**). Scale bar: 10 µm. **E** Immunoprecipitation (IP) of mCherry in HEK293S6 cells transfected with plasmids encoding HSP110-GFP, XPO1^WT^-mCherry, or XPO1.^E571K^-mCherry, followed by immunoblotting using anti-GFP, anti-mCherry, and anti-immunoglobulin heavy chains antibodies. An unrelated antibody was used as an IP control. **F** Quantification of the interactions of HSP110 with XPO1 in PMBL patients’ biopsies using Duolink technology; HSP110/Ig and XPO1/Ig bindings were used as negative controls. **G** Representative images of *in cellulo* interactions of HSP110-STAT6 as in (F). Scale bar: 50 µm. **H**, **I** Immunoblot analysis of HSP110 in the nucleus and in the cytosol in K1106P (**H**) and HEK293S6 cells (**I**) treated by selinexor (4 h, 1 µM). RelA, a known cargo protein of XPO1, was used as a positive control for selinexor effect. GAPDH was used as a cytosol loading control, and TBP (TATA binding protein) was used as a nuclear loading control. HSP110 and RelA protein levels are shown relative to the TBP or GAPDH for nucleus and cytosol respectively ns *P* > .05; * *P* < .05; ** *P* < .01; *** *P* < .001; **** *P* < .0001

XPO1 has been shown to be involved in the nuclear export of another heat shock protein (HSP70) in erythropoietic progenitors [44], and the specific XPO1 inhibitor selinexor is currently showing great efficacy in clinical trials. We therefore wondered if XPO1 was also involved in HSP110 nuclear export and cytosolic localization. We observed that XPO1 and HSP110 have a similar cytosolic localization in k1106P and MEDB1 cells (Fig. 2A and B) and we confirmed by PLA the proximity of both proteins (Fig. 2C and D). PLA with BRCA1 was used as a negative control. Although MedB1 expressed less HSP110 than K1106P (Fig. 1A), HSP110 and XPO1 showed a similar proximity. We transfected the HEK293 cell line with either XPO1^WT^ or XPO1^E571K^ plasmids together with HSP110 plasmid and showed by immunoprecipitation that HSP110 binds XPO1 independently of its mutation status (Fig. 2E). Furthermore, we used PMBL patient biopsies to show that XPO1 was strongly expressed (supplementary Fig. 1C) and that it interacts with HSP110 (Fig. 2F and G).

Treatment of K1106P by selinexor increases HSP110 nuclear localization, suggesting that HSP110 is a cargo of XPO1 (Fig. 2H). We proved this by expressing HSP110 in HEK293 cells and then treating them with selinexor. A higher nuclear localization was then observed (Fig. 2I). However, decreased expression of HSP110 by siRNA did not alter XPO1 protein expression, suggesting that HSP110 is not a chaperone of XPO1 (supplementary Fig. 1D).

### HSP110 inhibition synergizes with selinexor to reduce PMBL cell growth in vitro and in vivo

**Fig. 3:**
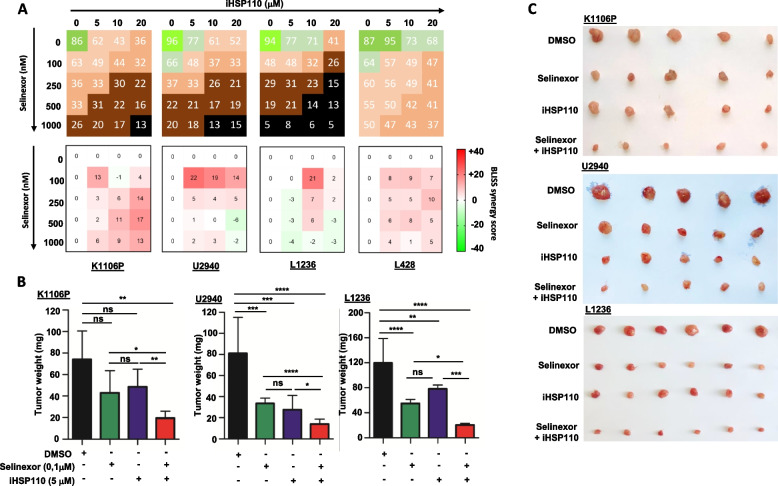
Synergistic decrease in PMBL and cHL cell survival and growth with HSP110 inhibitor and selinexor. **A** A drug dose matrix was established for K1106P, U2940, L1236, and L428 cell lines. Upper panel, the numbers within the matrix represent the percentage of cell growth with inhibitors (iHSP110-33 and selinexor) when used alone or in combination, relative to cells treated with just the compound DMSO (control). Data were color-coded and visualized in the matrix using a color scale. Lower panel, Bliss synergy score of inhibitors combination in K1106P, U2940, L1236 and L428. The numbers in the matrix indicate the antagonistic (below zero) or synergistic effect (above zero) of inhibitors (iHSP110 and selinexor) on cell growth (*n* = 3). **B** In vivo treatment with iHSP110-33 (5 µM) and selinexor (0.1 µM) synergistically reduces the tumor mass of K1106P, U2940 and L1236 cells in CAM of 17-day-old embryos. Tumor weights measured 7 days after xenograft. Mean mass (± SD) is presented (*n* = 5 per group). **C** Ex vivo images of representative K1106P, U2940 and L1236 xenograft tumors as in (B). ns *P* > .05; * *P* < .05; ** *P* < .01; *** *P* < .001; **** *P* < .0001

In search of combinational therapies, and given the interaction of HSP110 with XPO1, we investigated whether our HSP110 inhibitor could be associated with selinexor to obtain higher efficacy at lower concentrations. To achieve this goal, increasing doses of selinexor and iHSP110 were used in the three iHSP110-responsive cell lines shown in Fig. 1 (K1106P, U2940, L1236), and in the iHSP110-non-responsive L428 cell line. We observed a high inhibition of cell growth with the drug combination, together with a synergistic reduction in cell growth in all cell lines with low concentrations of both inhibitors. As expected, L428 was less responsive to the combination (Fig. 3A). The synergistic effect was lost in U2940 and L1236 cells at the highest concentration tested. We confirmed the efficacy of the combined treatment of selinexor and iHSP110, at concentrations found to synergize in vitro, in K1106P, U2940 and L1236 xenografts in chick chorioallantoic membranes. We observed tumor growth inhibition of 73% for K1106P with both inhibitors versus 42% for selinexor and 34% for iHSP110 monotherapy, 82% for U2940 with both inhibitors versus 58% for selinexor and 66% for iHSP110 monotherapy, and 82% for L1236 with both inhibitors versus 54% for selinexor and 34% for iHSP110 monotherapy (Fig. 3B, C).

**Fig. 4:**
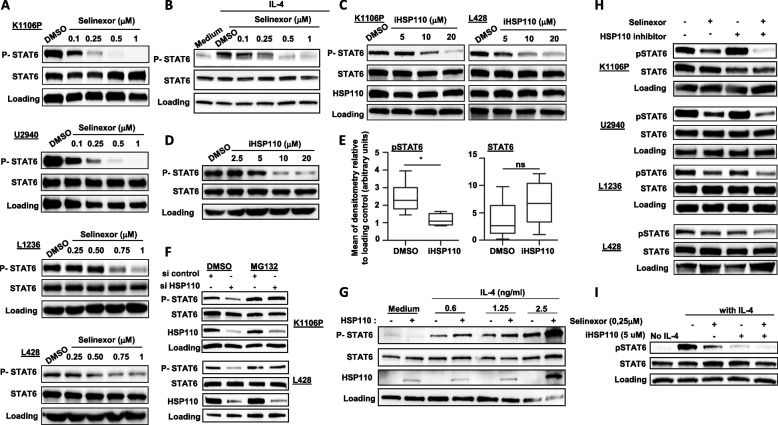
Synergistic decrease in STAT6 phosphorylation with HSP110-33 and selinexor combinational therapy. **A** Immunoblot analysis of P-STAT6 and STAT6 in K1106P, U2940, L1236 and L428 cells after 4 h treatment with increasing concentrations of selinexor or DMSO (control). **B** Immunoblot analysis of P-STAT6 and STAT6 in HEK293S6 cells after 24 h of IL-4 stimulation (0.6 ng/mL) and treatment with increasing concentrations of selinexor or DMSO (control) during the last 4 h. **C** Immunoblot analysis of P-STAT6, STAT6, and HSP110 in K1106P and L428 cells after 48 h treatment with increasing concentrations of iHSP110-33 or DMSO (control). **D** Immunoblot analysis of P-STAT6 and STAT6 in HEK293S6 cells after 24 h of IL-4 stimulation (0.6 ng/mL) and treated as in B. **E** Densitometry of P-STAT6 and STAT6 protein expression relative to the loading control from K1106P xenografted tumors from experiment in Fig. 1F (iHSP110-33 10 µM), (*n* = 7 per group). **F** Immunoblot analysis of P-STAT6, STAT6, and HSP110 in K1106P and L428 cells transfected with HSP110 siRNA or control siRNA, and treated by MG132 treatment (3 h, 10 µM) or control. **G** Immunoblot analysis of P-STAT6, STAT6, and HSP110 in HEK293S6 cells after 24 h of stimulation with increasing concentrations of IL-4, with or without transfection of a plasmid coding for HSP110-GFP. **H** Immunoblot analysis of P-STAT6 and STAT6 in K1106P, U2940, L428, and L1236 cells and (**I)** in IL-4-stimulated and HSP110-GFP transfected HEK293S6 cells treated with a combination of iHSP110-33 for 48 h (5 µM for the cells K1106P, L428 and HEK293S6); 10 µM for U2940 and 20 µM for L1236; and selinexor for 4 h (0.1 µM for K1106P); 0.25 µM for L428 and HEK293S6; and 0.5 µM for L1236. ns *P* > .05; * *P* < .05; ** *P* < .01; *** *P* < .001; **** *P* < .0001

To understand the mechanism of cell growth inhibition induced by the combination of iHSP110-33/selinexor, we analyzed the STAT6 signaling pathway, which is known to be a major driver of PMBL and cHL [6, 45, 46]. STAT6 is a known cargo of XPO1, and selinexor limits its cytosolic export [18], but the consequences of this nuclear retention on STAT6 activation is not known. We observed that selinexor induced a dose-dependent decrease in STAT6 phosphorylation in PMBL and cHL cell lines (Fig. 4A). We confirmed the inhibition of STAT6 activation by selinexor in HEK293 cells stably transfected with an expression vector coding for STAT6 (HEK293S6) and treated with IL-4 to induce the STAT6 signaling (Fig. 4B). We then wondered if HSP110 inhibition could also alter STAT6 signaling, since we previously showed that HSP110 has a role in STAT3 activation in colorectal cancer [41]. We observed that iHSP110-33 reduced, in a dose-dependent manner, the phosphorylation of STAT6 in PMBL and cHL cell lines as well as in HEK293S6 cells (Fig. 4C and D) and in vivo in K1106P tumor xenografts (Fig. 4E). This was confirmed when, instead of using our HSP110 inhibitor, we used a siRNA to downregulate HSP110 (Fig. 4F). The mechanism of inhibition was not mediated by a transcriptional effect but by a post-translational mechanism since it was blocked by a proteasome inhibitor, which led to the accumulation of P-STAT6 (Fig. 4F). Conversely, overexpression of HSP110 in HEK293S6 by plasmid transfection increased STAT6 phosphorylation in response to IL-4 stimulation (Fig. 4G). The nuclear localization of HSP110 was not necessary for this effect seeing as deletion of NLS had no impact on STAT6 phosphorylation (supplementary Fig. 1E). Finally, the presence of selinexor enhanced the ability of iHSP110-33 to reduce STAT6 phosphorylation (Fig. 4H and I). Altogether, these data show that HSP110 and XPO1 are essential for the STAT6 signaling pathway in PMBL and cHL.

### HSP110 is a chaperone for STAT6 and promotes its phosphorylation

Next, we explored how HSP110 could impact STAT6 phosphorylation. We previously showed that STAT6 is a cargo of XPO1, allowing the nuclear export [18]. We then wondered if the decrease of P-STAT6 could be due to alterations in STAT6-XPO1 binding. As shown in supplementary Fig. 2A, we confirmed the XPO1-STAT6 interaction and, using western blot densitometry analysis, observed similar proportions of STAT6 and XPO1 upon STAT6 immunoprecipitation in the presence of iHSP110 compared to the control. This data suggests that iHSP110 has no impact on STAT6-XPO1 interaction. Because STAT6 is phosphorylated in the cytosol near the plasma membrane, we determined whether iHSP110 could alter the cytosolic localization. As shown in supplementary Fig. 2B, the amount of total STAT6 in the cytosol was stable in the presence of iHSP110 while P-STAT6 decreased both in the cytosol and the nucleus. Thus, our data suggest that iHSP110 has no effect on STAT6 cytosolic export.

**Fig. 5:**
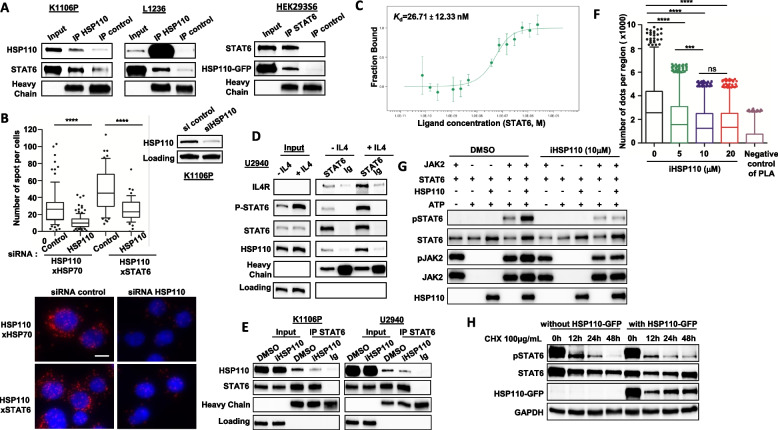
HSP110 chaperones STAT6 and promotes its phosphorylation. **A** Immunoprecipitation (IP) of HSP110 in K1106P, L1236, and of STAT6 in HEK293S6 cells, followed by immunoblot using anti-STAT6 for K1106P, and anti-GFP for HEK293S6. **B** Quantitation of HSP110xHSP70 and HSP110xSTAT6 interactions in K1106P in the presence of siRNA HSP110 or siRNA control. Representative images of in-cell interactions of HSP110xHSP70 and HSP110xSTAT6 are shown. Scale Bar: 10 µm. Immunoblot analysis of HSP110 knockdown is shown. B-actin served as a loading control. **C** Direct interaction study between fluorescently labeled HSP110 and STAT6 using microscale thermophoresis. HSP110 concentration was maintained at 50 nM, and STAT6 was titrated from 2.39 µM to 0.07 nM. The difference in normalized fluorescence [‰] was plotted for thermophoresis analysis. Error bars represent the standard error of 4 measurements. **D** Immunoprecipitation of STAT6 in U2940, stimulated or not for 30 min with IL-4, was followed by immunoblot using anti-IL-4R, anti-P-STAT6, anti-STAT6, anti-HSP110, and anti-immunoglobulin heavy chains. **E** Immunoprecipitation (IP) of STAT6 in K1106P and U2940 as in (D). Cells were treated with iHSP110-33 (20 µM) or DMSO as a control for 24 h prior to IP. **F** Quantification by Duolink technology of HSP110-STAT6 interaction in K1106P-derived tumor xenografts from experiment shown in Fig. 1. Negative control staining was performed with HSP110 antibody alone. **G** Immunoblot analysis of STAT6, P-STAT6, JAK2, P-JAK2, and HSP110 from an in vitro kinase assay in the presence of JAK2(50 ng), STAT6 (100 ng), with or without HSP110 (100 ng), ATP (250 µM), and in the presence or absence of iHSP110-33 (300 µM). **H** Immunoblot analysis of STAT6, P-STAT6, and GFP in HEK293S6 cells stimulated with IL-4 (0.6 ng/mL), with or without HSP110-GFP, and treated with cycloheximide (CHX) at 100 µg/mL for 12, 24, and 48 h. ns *P* > .05; * *P* < .05; ** *P* < .01; *** *P* < .001; **** *P* < .0001

To assess whether HSP110 chaperones STAT6, we started by performing immunoprecipitation experiments. We found that HSP110 binds to STAT6 in K1106P, L1236, HEK293S6 (Fig. 5A), U2940 (Fig. 5D) and MedB1 (supplementary Fig. 3A). We confirmed this interaction by PLA in K1106P (Fig. 5B) and MedB1 (supplementary Fig. 3B and C). HSP110-HSP70 interaction was used as a positive control. Using recombinant proteins and a MicroScale Thermophoresis assay, we demonstrated that STAT6 and HSP110 directly interacted with a *K*_d_ of 26.71 ± 12.33 nM (Fig. 5C). It is worth noting that HSP110-STAT6 interaction existed in the absence of exogeneous stimulation and was enhanced upon culture with IL4, which led to increased STAT6 phosphorylation in U2940 (Fig. 5D), and in K1106P and HEK293S (supplementary Fig. 4A). HSP110-STAT6 interaction was abrogated in the presence of iHSP110-33 as demonstrated by both immunoprecipitation (Fig. 5E and supplementary Fig. 4B) and PLA (Fig. 5F). In contrast, selinexor had no impact on HSP110-STAT6 interaction (supplementary Fig. 5). To get more insight into the mechanism of action, we performed an in vitro kinase assay with recombinant proteins. This assay showed that the total amount of STAT6 was increased in the presence of HSP110. HSP110 also enhanced STAT6 phosphorylation, whereas this phosphorylation was abrogated in the presence of the HSP110 inhibitor (Fig. 5G).

Furthermore, the study of the half-life of STAT6 in HEK293S cells overexpressing HSP110 or not, in the presence of the protein synthesis inhibitor cycloheximide, showed that HSP110 stabilized STAT6, which may contribute to its overall effect increasing STAT6 phosphorylation (Fig. 5H).

**Fig. 6:**
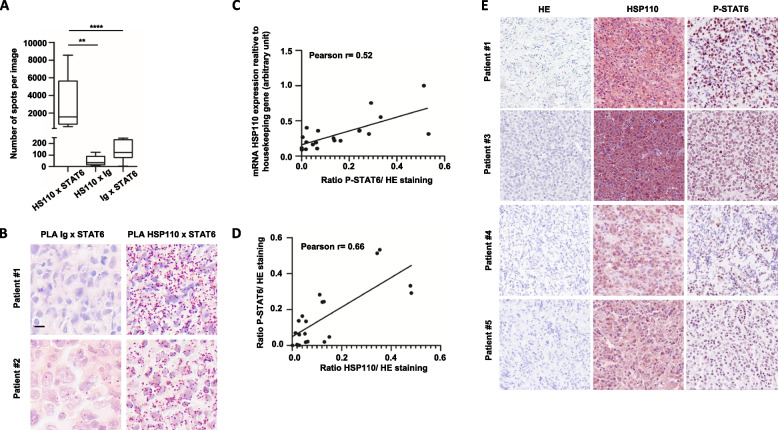
HSP110 expression correlates with STAT6 phosphorylation in PMBL patients. **A** Quantification of in situ interactions of HSP110 with STAT6 in PMBL patients’ biopsies using Duolink technology; HSP110xIg and STAT6xIg interactions were used as negative controls. **B** Representative images of *Duolink* HSP110-STAT6 interactions as in (A). Scale bar: 20 µm. **C** HSP110 mRNA expression intensity relative to HSP110 immunohistochemistry intensity in PMBL patients’ biopsies (*n* = 18). **D** HSP110 immunohistochemistry intensity relative to P-STAT6 immunohistochemistry intensity in PMBL patients’ biopsies (*n* = 18). **E** Representative images of HSP110 and P-STAT6 immunohistochemistry staining as in (C) and (D). ** *P* < .01; **** *P* < .0001

Finally, to substantiate the clinical relevance of these results, we analyzed the interaction between HSP110 and STAT6 by PLA in the biopsies of 18 PMBL patients. We confirmed a high level of HSP110-STAT6 interaction in all tumors (Fig. 6A and B). In addition, the level of HSP110, both mRNA (Fig. 6C) and protein (Fig. 6D and E), correlated with STAT6 phosphorylation (determined by IHC), further confirming the relationship between HSP110 and STAT6 activation in patients.

## Discussion

**Fig. 7:**
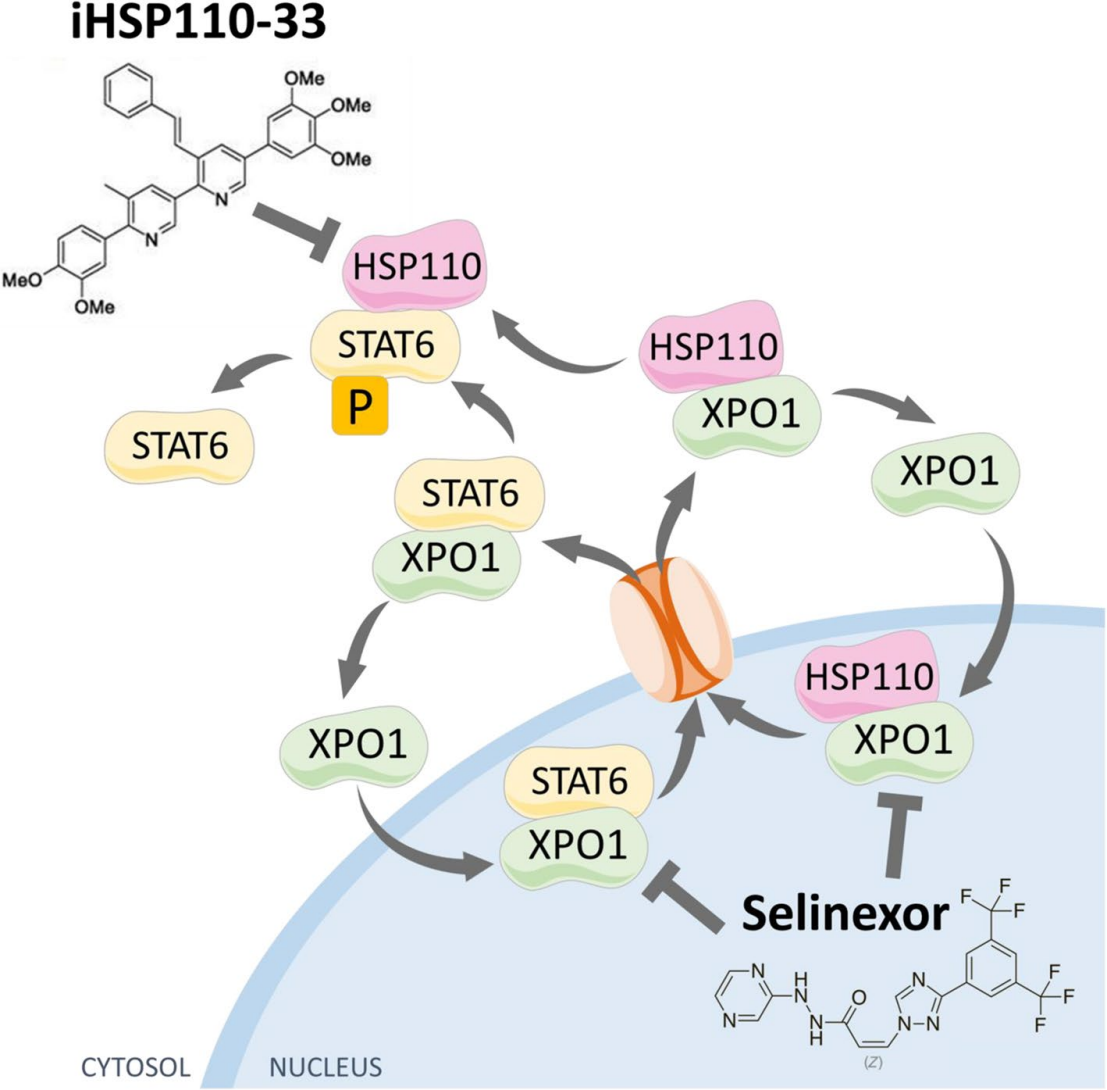
Proposed model for the role of HSP110 in the STAT6 signaling pathway and the combined selinexor and iHSP110 mechanisms of action. HSP110 and STAT6 are exported by XPO1 into the cytosol where HSP110 acts as a chaperone for STAT6 and promotes phosphorylation. Selinexor reduces HSP110 and STAT6 export. iHSP110 reduces STAT6 activation. The combination of iHSP110 and selinexor induces a synergistic reduction of STAT6 phosphorylation and of lymphoma cell growth

Though they have distinct B-cell origins, PMBL and cHL share multiple biological features such as the activation of the JAK/STAT signaling pathway leading to STAT6 dimerization and nuclear localization. As a result, almost 80% of PMBL and cHL patients have higher levels of phosphor-STAT6 in primary cells [45, 46]. Mutations in different components of the signaling pathway, such as SOCS1 and STAT6, and JAK gene amplification contribute to this activation [8–11]. Here, we show that HSP110 plays a central role in this machinery by stabilizing STAT6 and promoting its phosphorylation (Fig. 7). This function is concordant with what has been identified in colon cancer, in which HSP110 interacts with and activates STAT3 [41]. JAK2 amplification and SOCS1 mutation increase the whole STAT family, and it is very likely that HSP110 inhibition might not only reduce STAT6 but also STAT3 and STAT5 activity, resulting in stronger cell growth inhibition. Furthermore, other pathways such as NFkB are activated in cHL and PMBL, and we have shown previously that HSP110 sustains the activation of these pathways through MyD88 stabilization [33] The MyD88 mutation is absent in cHL and PMBL, but other HSPs have been implicated in the NFkB pathways in lymphomas, for instance HSP90, whose inhibition reduces Ikkα, β and δ [47]. Therefore, the involvement of HSP110 in other components of the NFkB pathway in cHL and PMBL would be worth determining. The oncogene BCL6 is transcriptionally repressed by P-STAT6 in PMBL cell lines [48], thus leading to a potential rise of its expression upon JAK/STAT inhibition. Fortunately, HSP110 is also a chaperone of BCL6, as shown in follicular lymphoma and Burkitt lymphoma [32], so HSP110 inhibition would prevent this bystander effect.

Although STAT inhibition does not systematically directly inhibit cell growth in cHL and PMBL, sensitization to current therapeutic agents such as vincristine and doxorubicine have been demonstrated [49]. HSP110 inhibition, by reducing activation of STAT family members or other currently unidentified pathways, would therefore act in a similar way and could be a facilitator for other therapies. With this rationale in mind, we explored HSP110 inhibition with the XPO1-targeting drug selinexor. First, we showed that XPO1 interacts with and exports HSP110 independently of the E571K mutation, which gives flexibility for cell targeting. The rate of HSP110 export in XPO1 ^E571K^ mutated cells is not known, but it could be affected like the rest of the exportome [19] and might have a consequence on the chaperoning capacity of HSP110. Sensitivity to iHSP110-33 is also E571K-independent, as demonstrated in cell lines that carry the mutated allele (MedB1 and L1236). Furthermore, K1106P (XPO1 wt) was sensitive whereas L428 (XPO1 wt) was resistant. This contrasts with other drugs like ibrutinib whose efficacy is improved by the E571K mutation in cHL and PMBL cell lines. Selinexor is approved for patients with relapsed and/or refractory (R/R) DLCBL or multiple myeloma [50, 51]. In most of these patients, selinexor shows significant efficacy but also causes adverse effects. Therefore, the search for combinational therapies with selinexor that could boost its efficacy, making it possible to reduce the dose of the drug and its toxicity, has been explored in various sub-types of NHL and cHL [26, 52–55]. The combination of iHSP110-33 with selinexor meets this objective because we show a synergistic effect with suboptimal concentrations of selinexor in all cHL and PMBL cell lines treated in vitro and in vivo.

Molecular characteristics, as seen in PMBL and cHL, should be more considered in the development of personalized medicine [56, 57]. Our study illustrates this idea considering that the effect of HSP inhibitors goes beyond the traditional lymphoma classification. We also suggest that therapeutic combinations involving an HSP110 inhibitor could be envisioned as a treatment for poorly understood lymphomas that share some similar molecular features with cHL and PMBL, such as gray zone intermediate lymphoma (MGZL) [58, 59]. Indeed, MGZL has been classified since 2022 by the WHO as a separate entity [39], being intermediate between cHL and PMBL.

## Conclusions

Our first-in-class inhibitor of HSP110 shows significant potential as a treatment against PMBL and cHL tumor growth when used alone or in combination with an XPO1 inhibitor (summarized in Fig. 7). We also believe that in the future it could be combined with other targeted therapies to offer alternative therapeutic options to patients.

### Supplementary Information

Supplementary Material 1.

Supplementary Material 2.

## Data Availability

Full materials, methods and data may be shared after request to the corresponding author by e-mail gaetan.jego@u-bourogne.fr.

## Abbreviations

BCL6: B-cell lymphoma 6
BRCA1: Breast cancer gene 1
CAM: Chorioallantoic membrane
cHL: Classical Hodgkin lymphoma
c-MYC: Cellular-Myelocytomatosis oncogene
DLBCL: Diffuse large B-cell lymphoma
HRS: Hodgkin and Reed–Sternberg
HSP: Heat-shock protein
Nhibitor: Of HSP110
IKBKB: Inhibitor of NFKB kinase subunit Beta
JAK: Janus kinase
LBCL: Large B-cell lymphoma
MYD88: Myeloid differentiation primary response 88
NFkB: Nuclear factor-kappa B
NFKB2: NFKB subunit 2
NFKBIE: NFKB inhibitor Epsilon
NSCHL: Nodular sclerosis classical Hodgkin lymphoma
PMBL: Primary mediastinal B-cell lymphoma
PTPN1: Protein tyrosine phosphatase, non-receptor type 1
SOCS1: Suppressor of cytokine signaling 1
STAT: Signal transducers and activators of transcription
TNFAIP3: Tumor necrosis factor, a-induced protein 3
XPO1: Exportin 1
