# Supplementary material for: A first-in-class inhibitor of HSP110 to potentiate XPO1-targeted therapy in primary mediastinal B-cell lymphoma and classical Hodgkin lymphoma
Source: J Exp Clin Cancer Res. 2024 May 22;43:148. doi: 10.1186/s13046-024-03068-x (PMC11110392; doi:10.1186/s13046-024-03068-x)
Supplement: Supplementary file 1 — Supplementary Material 1. [file 13046_2024_3068_MOESM1_ESM.pdf]

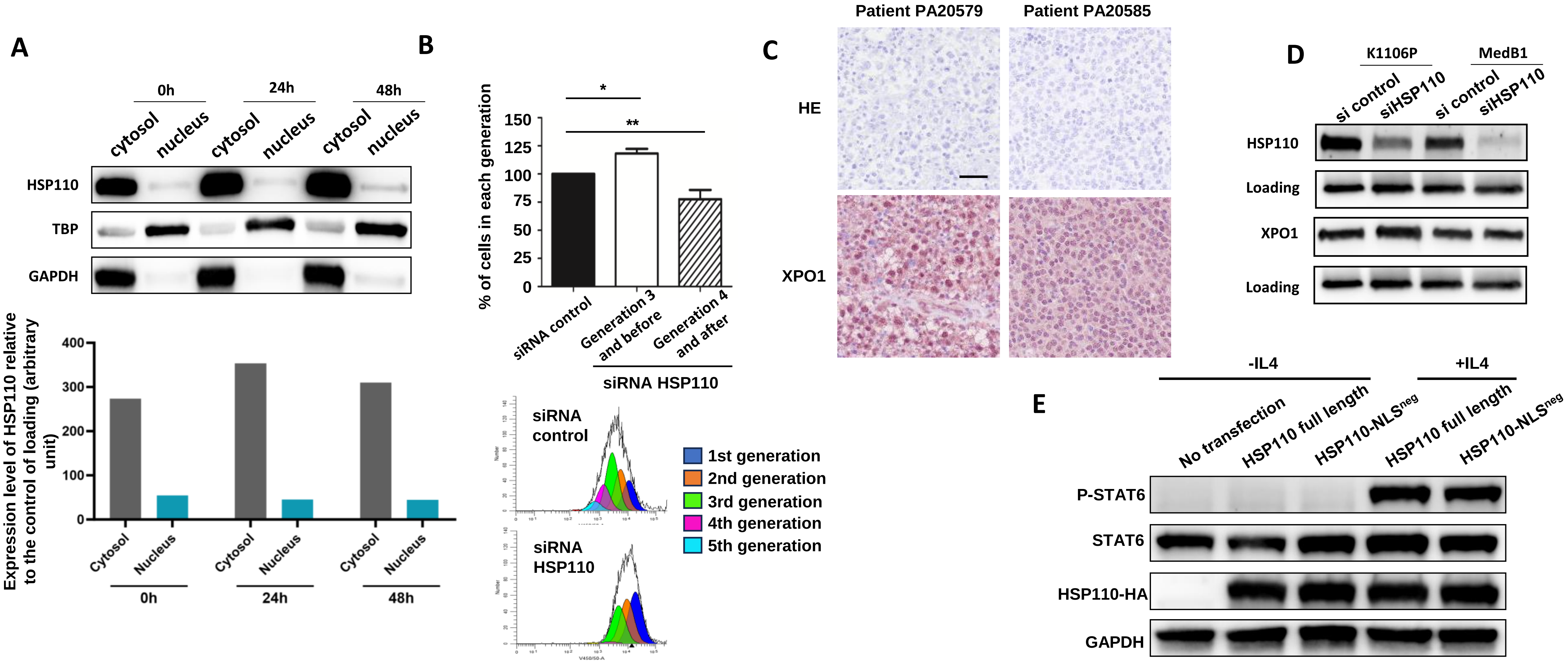

(A) Immunoblot analysis of cytosolic and nuclear HSP110, TBP (TATA binding protein as a nuclear specific loading control) and GAPDH in K1106P, 24 and 48h after treatment with iHSP110-33. HSP110 protein level is shown relative to the TBP or GAPDH for nucleus and cytosol respectively. (B) Percentage of K1106P cells in each cell division 72h after transfection with HSP110 siRNA (n=3). One representative flow cytometry histogram is shown below. Proliferation was assessed as dilution of CellTrace fluorescent dye by flow cytometry. (C) representative image of XPO1 expression in two PMBL patients. HE is hematoxylin-eosin staining. Scale bar: 50 µm. (D) Immunoblot analysis of HSP110 and XPO1 in K1106P and MedB1 48h after transfection with a HSP110 siRNA. (E) Immunoblot analysis of P-STAT6, STAT6, HSP110-HA and GAPDH in HEK293S 48h after transfection with a HSP110-HA full-length or HSP110-HA without nuclear localization signal (HSP110-NLS ^{neg}), with or without IL4. GAPDH was used as a loading control.

Figure 1 sup

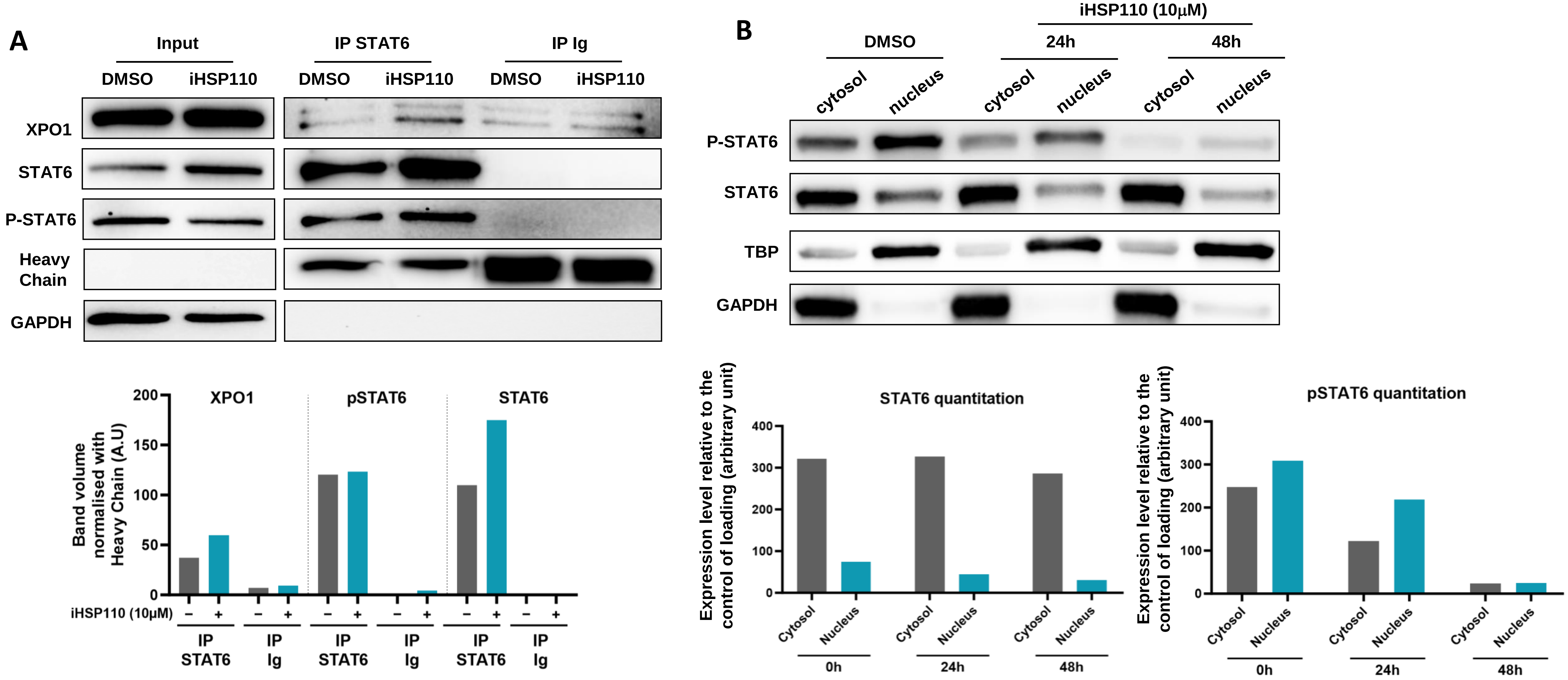

(A) Immunoprecipitation (IP) of STAT6 in K1106P cells treated with 10µM iHSP110-33 during 48h followed by immunoblotting using anti-XPO1, anti-HSP110, anti-pSTAT6, anti-STAT6, anti-GAPDH, and anti-immunoglobulin heavy chains antibodies. An unrelated antibody was used as an IP control. Densitometry of XPO1, STAT6, and P-STAT6 from the above western blot is shown relative to the level of heavy chains. (B) Immunoblot analysis of P-STAT6, STAT6 in the cytosol an in the nucleus in K1106P after treatment for 24 hours and 48 hours with 10µM of iHSP110-33. GAPDH was used as a cytosol loading control, and TBP (TATA Binding Protein) was used as a nuclear loading control. Densitometry of STAT6, P-STAT6 from the above western blot, is shown relative to the level of the TBP or GAPDH for nucleus and cytosol respectively.

Figure 2 sup

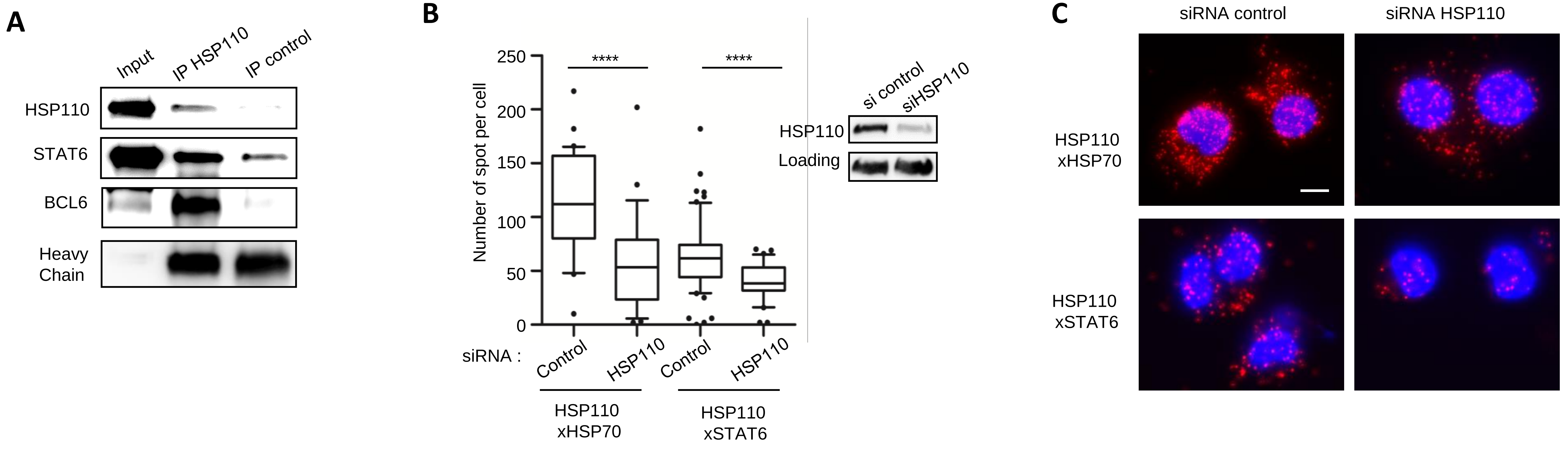

(A) Immunoprecipitation (IP) of HSP110 in MedB1, followed by immunoblot using anti-HSP110, anti-STAT6, anti-Bcl6 and anti-immunoglobulin heavy chains. A non-relevant antibody was utilized as a control (IP control). (B) Quantitation of HSP110xHSP70 and HSP110xSTAT6 interactions in MedB1 using Duolink technology in the presence of siRNA HSP110 or siRNA control. HSP70 was used as a positive control for HSP110 interaction. (C) Representative images of in-cell interactions of HSP110xHSP70 and HSP110xSTAT6 are shown. Scale Bar: 10 µm ****P < .0001

Figure 3 sup

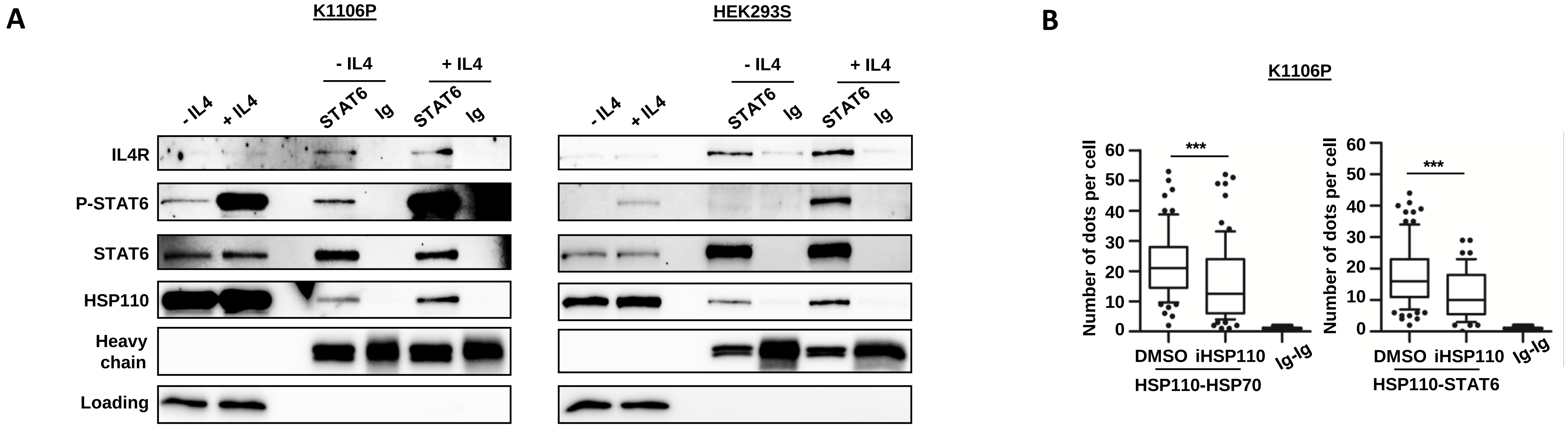

(A) Immunoprecipitation of STAT6 in K1106P (left) and in HEK293S6 (right), followed by immunoblot using anti-IL4R, anti-P-STAT6, anti-STAT6, anti-HSP110, and anti-immunoglobulin heavy chains. Anti-vinculin antibody served as loading control. A non-relevant antibody was used as a control (Ig). Cells were IL4 stimulated or not for 30min prior to IP. (B) Quantitation of HSP110-HSP70 and HSP110-STAT6 interactions in K1106P using Duolink technology in the presence of siRNA HSP110 or siRNA control. HSP70 was used as a positive control for HSP110 interaction.

Figure 4 sup

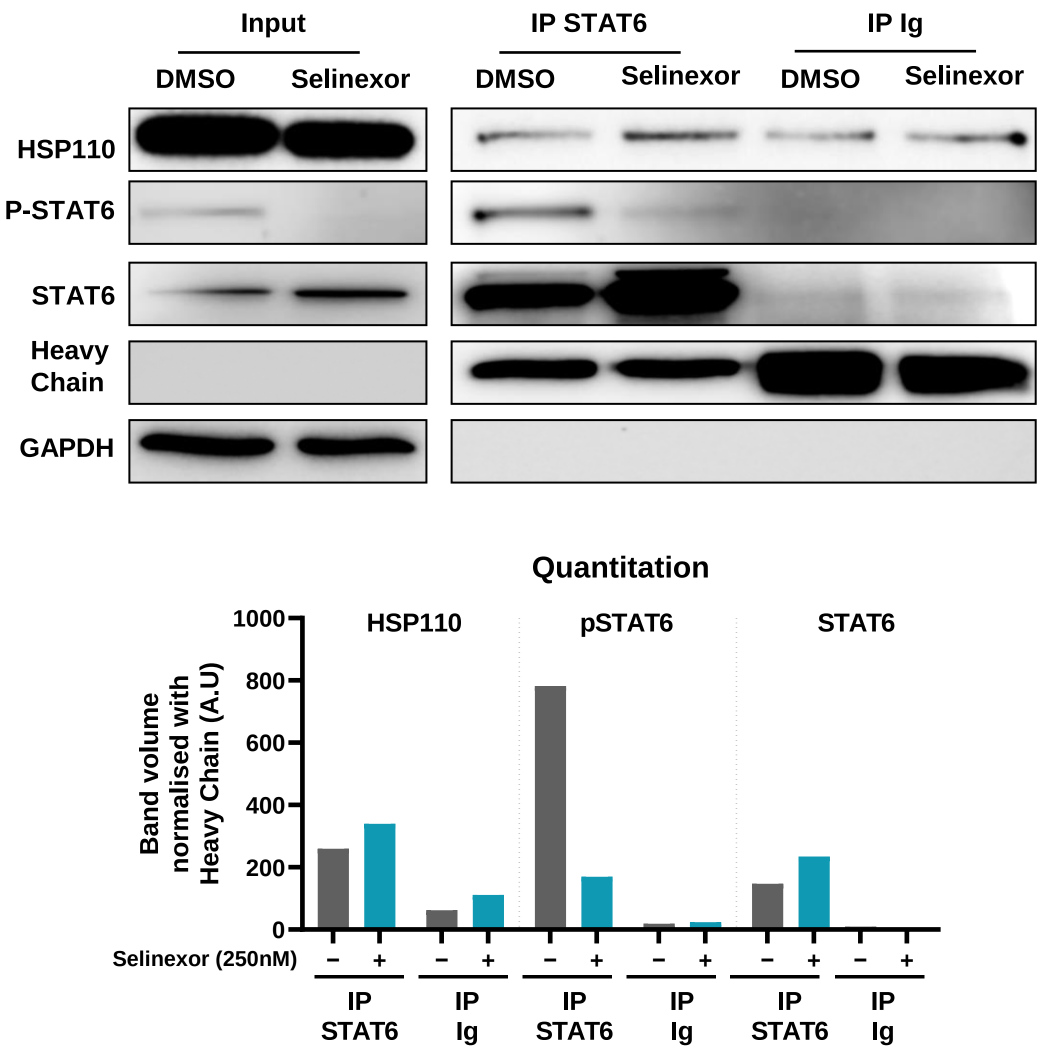

Immunoprecipitation (IP) of STAT6 in K1106P cells transfected treated with selinexor (250nM) during 24h followed by immunoblotting with anti-HSP110, anti-P-STAT6, anti-STAT6, anti-GAPDH, and anti-immunoglobulin heavy chains antibodies. An unrelated antibody was used as an IP control (IP Ig). Densitometry of HSP110, STAT6, P-STAT6 from the above western blot, is shown relative to the level of heavy chains.

Figure 5 sup
