# Supplementary material for: A first-in-class inhibitor of HSP110 to potentiate XPO1-targeted therapy in primary mediastinal B-cell lymphoma and classical Hodgkin lymphoma
Source: J Exp Clin Cancer Res. 2024 May 22;43:148. doi: 10.1186/s13046-024-03068-x (PMC11110392; doi:10.1186/s13046-024-03068-x)
Supplement: Supplementary file 2 — Supplementary Material 2. [file 13046_2024_3068_MOESM2_ESM.pdf]

Target	Supplier	Reference	Dilution
**STAT6**	Cell Signaling Technology	5397S	1:1000(WB) or 1:50 (IP for 250µg -1mg of total protein)
**pSTAT6**	Cell Signaling Technology	9361S	1:1000(WB)
**XPO1**	Cell Signaling Technology	46249S	1:1000 (WB)
**GAPDH**	Santa Cruz Biotechnology	sc-47724	1:1000 (WB)
**Vinculin**	Sigma-Aldrich (Merck Group)	V9131	1:1000 (WB)
**GFP**	Abcam	ab290	1:1000 (WB)
**mCherry**	Abcam	ab213511	1:1000 (WB) or 1/20 (IP for 250µg -1mg of total protein)
**HSP60**	Cell Signaling Technology	12165S	1:1000 (WB)
**RelA**	Cell Signaling Technology	8242S	1:1000 (WB)
**TBP (TATA Binding Protein)**	Cell Signaling Technology	D5C9H	1:1000 (WB)
**Lamin A**	Thermo-Scientific	MA3-1000	1:1000 (WB)
**HSP110**	Santa Cruz Biotechnology	sc-74550	1:1000 (WB) or 1:100 (IP for 250µg -1mg of total protein)
**anti-rabbit IgG HRP-linked**	Cell Signaling Technology	7074S	1:5000 (WB)
**anti-mouse IgG HRP-linked**	Cell Signaling Technology	7076S	1:5000 (WB)

Supplementary Table 1 : Dilution and reference of the antibodies used for Western Blot (WB) and immunoprecipitaion (IP)
